# Leveraging geographic information system for dengue surveillance: a scoping review

**DOI:** 10.1186/s41182-025-00783-9

**Published:** 2025-08-04

**Authors:** Prathiksha Prakash Nayak, Jagadeesha Pai B., Sreejith Govindan

**Affiliations:** 1 Department of Civil Engineering, Manipal Institute of Technology, Manipal Academy of Higher Education, Manipal, 576104 India; 2 Division of Microbiology, Department of Basic Medical Sciences, Manipal Academy of Higher Education, Manipal, 576104 India

**Keywords:** Geographic information systems, Disease surveillance, Spatial analysis, Dengue, Vector-borne diseases, Public health

## Abstract

**Background:**

Vector-borne diseases caused by *Aedes* mosquitoes remain a major public health concern across tropical and subtropical regions. Geographic Information Systems (GIS) have become integral in surveillance by enabling spatial analysis, risk mapping, and predictive modelling. This scoping review explores how GIS has been applied in surveillance studies and identifies its potential applications, key variables, modelling approaches, and challenges.

**Methods:**

This scoping review was conducted following PRISMA-ScR guidelines and was structured using a search strategy to identify relevant peer-reviewed articles published between 2015 and 2024 across databases like PubMed, Scopus, ScienceDirect, and Google Scholar. A total of 64 studies were selected and charted based on geographic focus, GIS applications, modelling techniques, spatial methods, and key variables.

**Results:**

A notable concentration of studies was found in South and Southeast Asia, reflecting the high disease burden and research activity in these regions. ArcGIS and QGIS were the most frequently used platforms in dengue surveillance around the globe. Risk mapping and hotspot detection were the most frequent targeted applications (n = 26), followed by vector control and monitoring (n = 23). Environmental and climatic variables were commonly analysed, including temperature, rainfall, humidity, and Normalised Difference Vegetation Index. Common analytical methods included regression-based spatial models and, increasingly, machine learning techniques along with GIS. Emerging trends include integrating machine learning models, remote sensing data, and mobile GIS for real-time monitoring and early warning systems.

**Conclusions:**

GIS has evolved from a mapping tool into a multidimensional decision-support system in disease surveillance. Its integration with environmental, climatic, and demographic data enables proactive outbreak management and targeted interventions. Future research should leverage Artificial Intelligence, machine learning, the Internet of Things, participatory GIS, and interdisciplinary data to enhance surveillance prediction and public health response. Strengthening collaborative data-sharing frameworks and incorporating machine-learning approaches could further improve the effectiveness of GIS-driven surveillance programs.

**Supplementary Information:**

The online version contains supplementary material available at 10.1186/s41182-025-00783-9.

## Introduction

Dengue fever, caused by the *Aedes aegypti* and *Aedes albopictus* mosquito species, has emerged as one of the most critical vector-borne diseases globally, affecting millions annually [1, 2]. With an estimated 390 million infections, of which 96 million are clinically evident, dengue challenges healthcare systems in endemic regions, particularly tropical and subtropical climates [3]. The World Health Organisation (WHO) estimates that approximately half of the world’s population is at risk of dengue infection, with about 100–400 million infections occurring annually. Countries such as India, Nepal, and Bangladesh have experienced significant dengue outbreaks in Asia. In India, states such as Kerala and northeastern regions bordering Bangladesh reported increased cases in 2023 compared to previous years [4]. In 2024, over 14 million dengue cases and more than 10,000 dengue deaths were reported globally. The disease imposes a significant economic burden on healthcare resources and communities through lost productivity and long-term health complications. Factors such as increasing urbanisation, inadequate waste management, and evolving climate patterns have further intensified the spread of the disease, facilitating the proliferation of its vectors. This necessitates adopting innovative, multidisciplinary approaches for effective monitoring and control [2, 5].

The primary advantage of GIS lies in its ability to map and analyse spatial patterns of disease incidence. This facilitates the identification of high-risk areas or hotspots, which are critical for targeted interventions. Moreover, GIS enables the integration of diverse data sets, such as meteorological factors (e.g., temperature, rainfall, and humidity), land use/land cover (LULC), and socio-demographic variables (e.g., population density and urbanisation rates) [6, 7]. These factors are vital in understanding mosquito breeding habitats, human–vector interactions, and the overall risk landscape. Such integrated analyses are crucial for designing effective vector control measures, optimising resource allocation, and prioritising areas for intervention [8].

Beyond mapping, advancements in predictive modelling have further expanded the applications of GIS in dengue surveillance. Risk mapping, assessment, and outbreak forecasting have gained prominence, leveraging methodologies, such as spatiotemporal clustering, machine-learning algorithms, and multi-criteria decision analysis (MCDA) [2, 9–12]. These techniques enable the development of models that can accurately predict outbreak patterns, incorporating variables, such as vegetation indices derived from satellite imagery, rainfall patterns, and temperature fluctuations [1, 12]. Predictive models enhance outbreak preparedness and support public health officials in implementing timely and effective interventions.

GIS has emerged as a transformative tool, enabling the spatial and temporal analysis of disease dynamics [13]. By integrating epidemiological, environmental, and socio-demographic data, GIS offers a comprehensive platform to explore the complex interplay of factors driving dengue transmission [14].

The integration of socio-economic data, such as access to healthcare, sanitation, and community awareness levels, remains underexplored in many studies. These gaps underscore the need for more inclusive data sets and interdisciplinary collaboration to refine existing models and make them more actionable. This scoping review synthesises the findings of 64 studies to provide a comprehensive understanding of the applications of GIS in dengue surveillance. It explores the diverse methodologies employed, such as risk mapping and predictive modelling, while examining the integration of environmental, ecological, and socio-economic factors. The review aims to highlight the transformative potential of GIS in enhancing surveillance monitoring, identifying its role in dengue surveillance, key variables, modelling approaches, and proposing future directions for improving surveillance strategies.

## Materials and methods

**Fig. 1:**
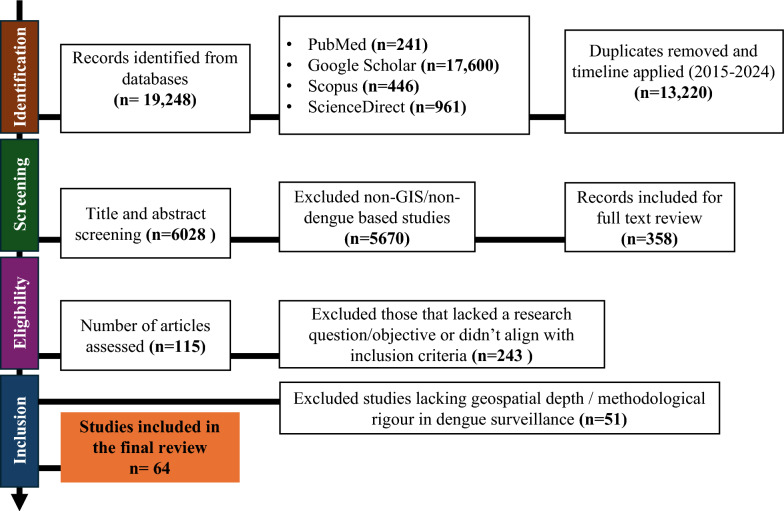
PRISMA flow diagram

This scoping review followed the framework proposed by Arksey and O’Malley and adhered to the PRISMA–ScR (Preferred Reporting Items for Systematic Reviews and Meta-Analyses Extension for Scoping Reviews) guidelines to ensure a transparent and comprehensive assessment of the literature (Fig. 1).

### Research question

1. How has GIS been applied in dengue, and what spatial techniques are commonly used?
2. What are the key risk factors identified through GIS-based analysis in dengue surveillance?
3. What challenges and future directions exist in utilising GIS for dengue monitoring?

### Identifying relevant studies

A comprehensive literature search was conducted across PubMed, Scopus, ScienceDirect and Google Scholar using a search strategy: (“Dengue” OR “Dengue fever” OR “Dengue virus” OR “*Aedes aegypti*” OR “*Aedes albopictus*”) AND (“Geographic Information System” OR “GIS” OR “Spatial analysis” OR “Geospatial analysis” OR “Mapping”) AND (“Surveillance” OR “Disease monitoring” OR “Vector surveillance” OR “Outbreak prediction” OR “Risk mapping”).

### Study selection

1. Title and abstract screening—screened studies on GIS-based dengue surveillance.
2. Full-text review—Articles meeting the criteria underwent full-text evaluation to ensure methodological and thematic alignment with the study objectives/research question, considering inclusion/exclusion criteria.
3. Inclusion criteria:
  Publication type: Peer-reviewed journal articles, conference publication.
  Time frame: Published between 2015 and 2024.
  Language: Published in English.
  Geographic focus: Studies from any global region.
  Content focus: Studies that used GIS tools or geospatial methods/applications in dengue surveillance.
  Study types: Both original research that explicitly applies or discusses GIS in dengue surveillance.
4. Exclusion criteria:
  Language: Articles not published in English.
  Irrelevant focus: Studies that did not include GIS or spatial analysis as part of dengue surveillance.
  Disease scope: Studies focused on other diseases (e.g., malaria, chikungunya) unless dengue-specific GIS insights were presented.
  Lack of access or quality: Articles without full-text availability or that lacked sufficient methodological detail.
  Duplicates: Studies duplicated across databases were removed.
  Editorials, opinions, conference publication or commentaries: These were excluded unless they presented a GIS-based framework or methodological insight.

### Charting data

Studies that stated a research question or objective with the mentioned inclusion criteria were selected to assess methodological clarity. Relevant information from the selected studies was systematically extracted and organised into a structured data chart. Key details, including study objectives, geographic region of study, GIS methodologies, GIS software, modelling techniques, use of remote sensing, dengue surveillance focus, spatial analysis techniques, risk factors, significant findings and limitations were recorded to facilitate thematic analysis and synthesis. These domains included: (1) risk mapping and hotspot identification, (2) vector control and monitoring, (3) urban and environmental planning, (4) early warning systems (EWS), (5) disease surveillance and public health monitoring, and (6) resource allocation and healthcare planning. Given the multifocal nature of the included studies, each was allowed to be classified under multiple GIS application domains based on its objectives, analyses, and outcomes.

Of the 115 articles initially screened, 64 met the final inclusion criteria after removing duplicates, excluding non-GIS-based studies, and assessing full-text relevance to dengue surveillance using geospatial tools. Studies lacking methodological detail, having limited spatial analysis, or focusing solely on non-dengue contexts were excluded after full-text review.

### Collating, summarising and reporting results

**Fig. 2:**
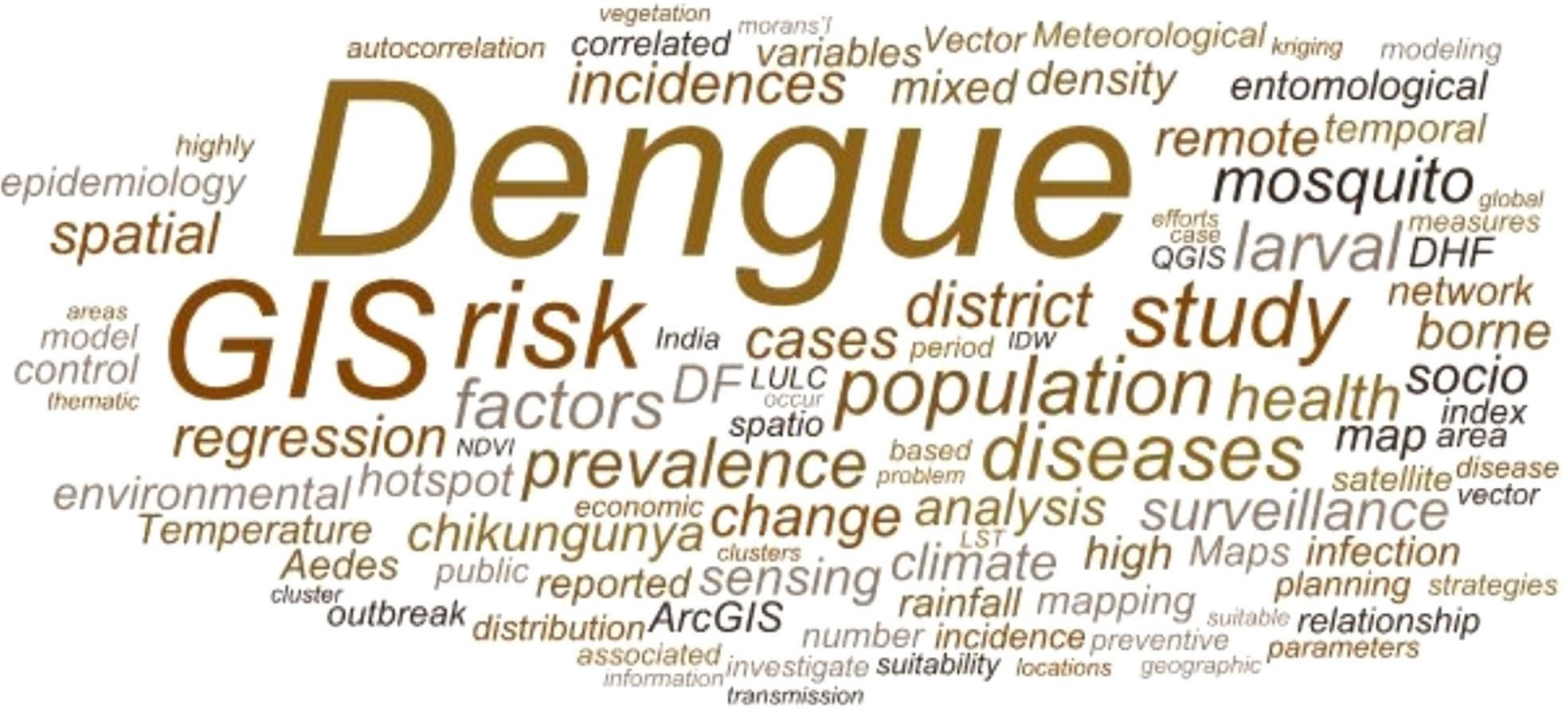
Word cloud

Data were synthesised quantitatively (distributions of GIS techniques and applications) and qualitatively (key insights). A word cloud was also produced to detect keywords that appeared often in the abstracts of the publications reviewed. To determine the most popular keywords in GIS-based dengue surveillance studies, the abstracts of all articles were examined. The word cloud graph displays the top 100 words from the abstracts of the 64 articles that were chosen, shown in Fig. 2.

## Results

### Overview of included studies on GIS-based dengue surveillance

**Fig. 3:**
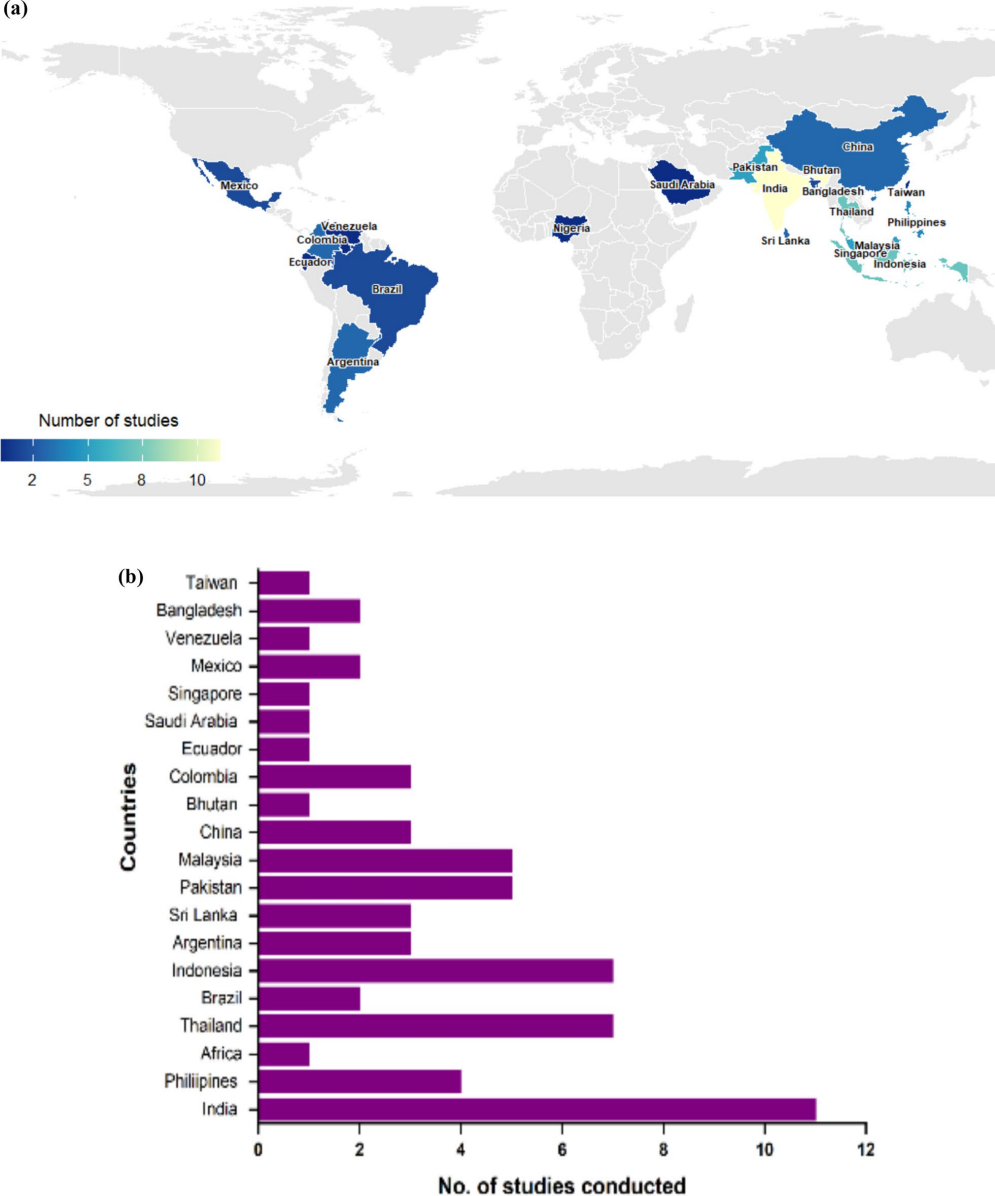
**A** Geographic distribution of GIS-based dengue surveillance studies. **B** Geographic distribution of GIS-based dengue surveillance studies

The included studies observed the widespread application of GIS in dengue across diverse geographic regions. The 64 studies reviewed were multifocal in scope, with many addressing more than one GIS application area, such as risk mapping, vector control, early warning systems, and urban planning. Many studies were conducted in dengue-endemic areas, particularly in South and Southeast Asia, including India, Thailand, and Indonesia (Fig. 3A, B) [15–18]. Studies in South America and Brazil focused on predictive modelling and dengue case distribution analysis [19, 20]. GIS tools such as ArcGIS, QGIS, and Google Earth Engine were frequently used, with ArcGIS being the most utilised (48%) for spatial analysis, due to its advanced spatial analysis tools, seamless remote sensing integration, and strong technical support [21, 22]. QGIS (18%) was used in studies favouring open-source alternatives [18, 23]. However, several studies did not specify the GIS tools used for mapping and spatial analysis, limiting transparency and reproducibility.

Remote sensing applications were integrated into approximately 30% of the studies, providing crucial environmental data, such as bioclimatic variables, elevation, and land use patterns [24]. Remote sensing data from satellites such as MODIS (moderate resolution imaging spectroradiometer), Landsat, and Sentinel have been incorporated to monitor environmental changes that precede dengue outbreaks, such as increased vegetation or stagnant water bodies that serve as breeding grounds for mosquitoes [25–27].

**Fig. 4:**
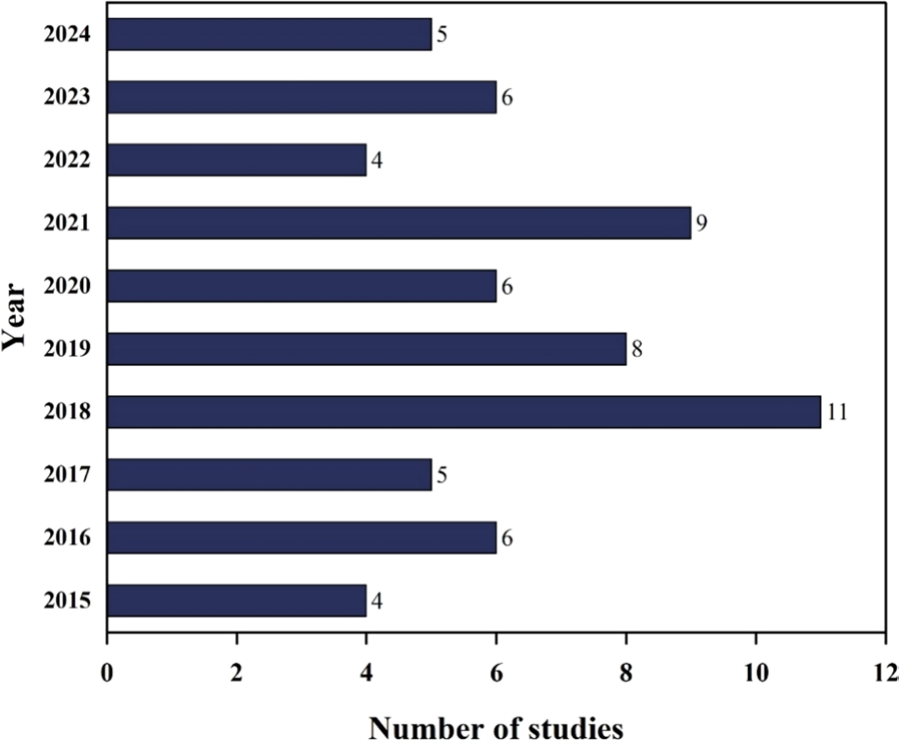
Yearwise trend of studies

NDVI and Land Surface Temperature (LST) are widely used in remote sensing for dengue surveillance studies, because they strongly indicate mosquito habitat suitability [28]. NDVI helps identify vegetation density, which correlates with mosquito breeding and resting sites, while LST reflects surface temperature, influencing mosquito development and virus transmission [29, 30]. Using satellite imagery and indices such as NDVI and LST helped researchers identify high-risk zones and breeding habitats, particularly in urban areas, where traditional surveillance is challenging [26, 31]. The integration of these technologies facilitated high-resolution dengue risk mapping, allowing public health authorities to implement targeted vector control strategies. The highest number of studies was published in 2018, possibly due to increased research interest in GIS-based disease mapping advancements using remote sensing applications. It heightened global attention to climate-driven disease outbreaks, as shown in Fig. 4.

### Common GIS applications in GIS-based dengue surveillance studies

**Fig. 5:**
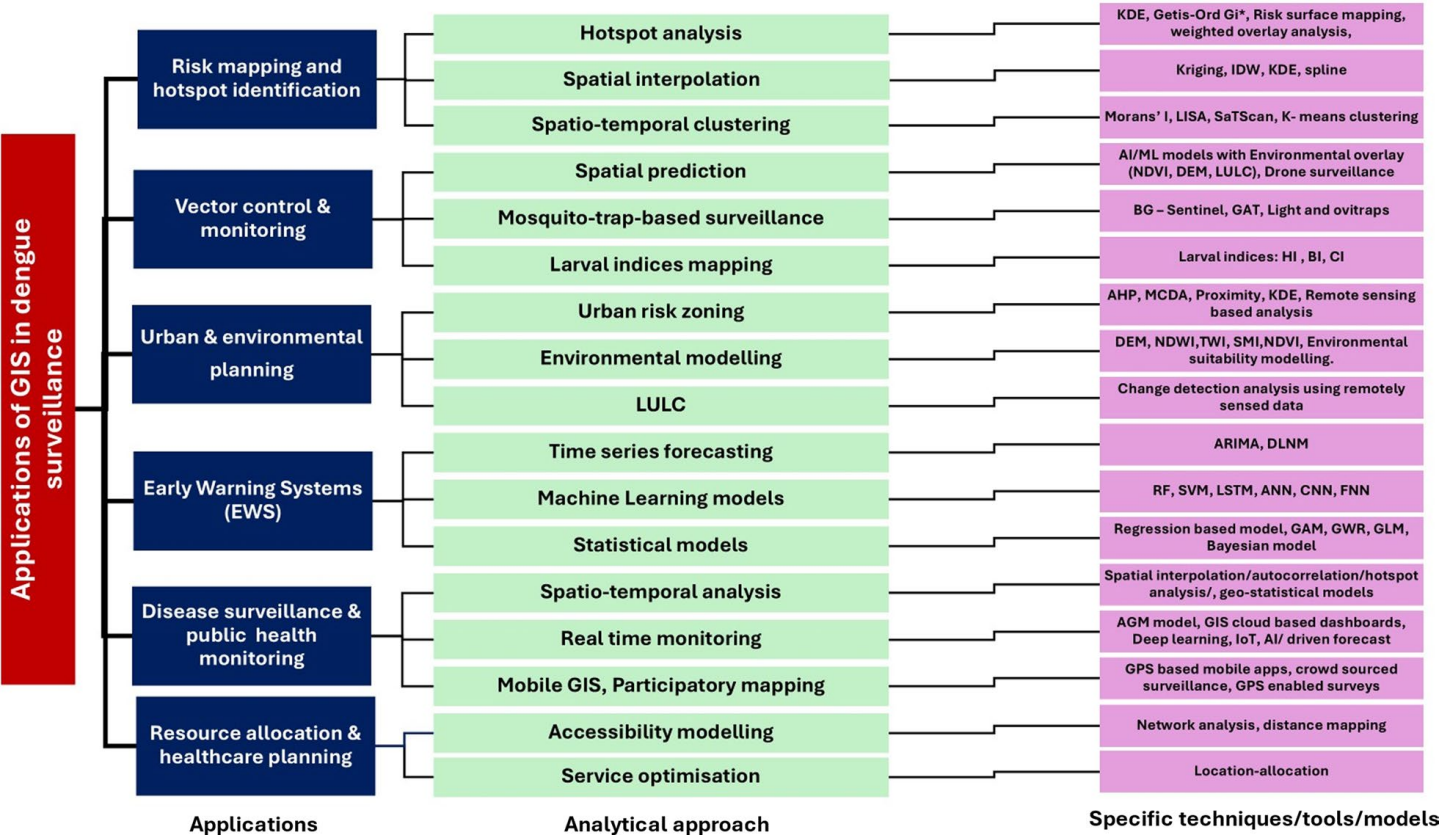
Taxonomy chart for GIS methods and applications in dengue surveillance

**Fig. 6:**
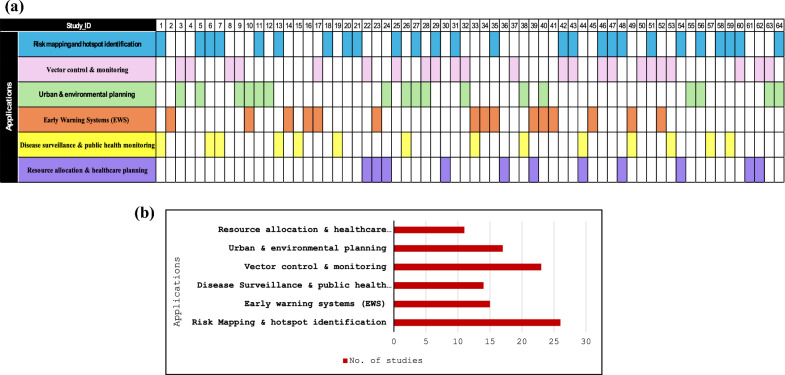
**A** Multifocal applications of GIS-based dengue surveillance studies. **B** GIS-based potential applications in dengue surveillance

The taxonomy chart, i.e., Fig. 5, presents a structured hierarchical classification of GIS applications in dengue surveillance. Figure 6A illustrates a matrix categorising studies with multifocal applications of GIS in dengue surveillance. Studies appearing in more than one category are represented across multiple cells, with their study IDs referenced in the Supplementary Material.

#### Risk mapping and hotspot identification

One of the most extensively explored applications of GIS in dengue surveillance is risk mapping and hotspot identification, which plays a crucial role in understanding the spatial distribution of disease outbreaks. Risk mapping and hotspot analysis were employed in a total of *n* = 26 of the reviewed studies, as observed in Fig. 6A, B. By integrating epidemiological data with environmental and climatic factors, GIS-based mapping techniques provide a visual representation of high-risk areas, enabling targeted interventions [21, 32, 33]. Several studies have employed hotspot analysis techniques, such as Local Indicators of Spatial Association (LISA), Getis-Ord Gi and Moran’s I [34–37]. These methods help identify statistically significant high-incidence zones, where disease transmission is persistent over time. Kernel density estimation (KDE) and spatial interpolation models such as inverse distance weighting (IDW) and kriging have been used to generate continuous risk surfaces, allowing public health officials to predict areas prone to future outbreaks [30, 33, 38, 39]. Integrating remote sensing data, including LST, vegetation indices, and LULC classifications, further enhances the accuracy of these models by correlating dengue hotspots with environmental suitability for vector breeding.

GIS-based hotspot analysis, mapping (Getis-Ord Gi, Moran’s I, and KDE) helps detect persistent mosquito activity zones, ensuring that control measures such as insecticide spraying, larval source management, and environmental sanitation campaigns are spatially targeted rather than randomly deployed [37, 40, 41]. Other techniques for hotspot analysis include Spatial Scan Statistics (SaTScan) for detecting clusters, Spatiotemporal clustering (STC) for tracking outbreaks over time, Hierarchical clustering for grouping risk zones, and Point pattern analysis (PPA) for identifying case distribution patterns [18, 25]. However, these methods are less commonly used due to their computational complexity, high data requirements, and limited accessibility in public health settings. SaTScan and STC rely on precise temporal data, which may not always be available, while hierarchical clustering and PPA may lack the spatial precision needed for targeted interventions.

MCDA is a decision-support framework that evaluates multiple spatial factors such as land use, population density, and climatic conditions to identify priority areas for vector control and resource allocation [42]. By systematically weighing and ranking different risk factors, MCDA enhances the precision of dengue risk mapping. When integrated with GIS, this approach enables the creation of detailed risk maps incorporating environmental and socio-economic variables, allowing for more accurate identification of high-risk zones. These maps assist public health officials in optimising resource distribution and implementing targeted interventions. The study underscores the potential of combining MCDA and GIS to improve vector-borne disease surveillance and control strategies [9]. Using GIS in risk mapping and hotspot identification has proven instrumental in proactively addressing dengue transmission, helping authorities deploy preventive measures in areas of highest vulnerability [43].

#### Vector control and monitoring

GIS has played a crucial role in vector control and monitoring, enabling researchers and public health authorities to identify mosquito breeding habitats, optimise control strategies, and track vector populations [44, 45]. Among all the studies reviewed, *n* = 23 focused on vector control and monitoring**,** emphasising the effectiveness of GIS in mapping high-risk zones, predicting mosquito proliferation, and guiding targeted interventions (Supplementary Table 1). GIS-based approaches facilitate spatial analysis of larval indices, including the House Index (HI), Breteau Index (BI), and Container Index (CI), to assess vector density across urban and rural landscapes [37, 44, 46].

Spatial interpolation techniques such as Kriging, IDW and spline have been extensively used to predict mosquito breeding hotspots based on historical occurrences and environmental factors [39, 47–49]. Remote sensing applications, including NDVI and LULC classification, have further enhanced vector monitoring by identifying potential breeding sites, particularly in areas with stagnant water, poor drainage, and dense vegetation [28]. Drone-assisted GIS mapping would improve surveillance by capturing high-resolution images of inaccessible breeding sites, ensuring comprehensive monitoring and intervention planning.

GIS has also been instrumental in strategically placing and analysing mosquito traps, allowing for real-time vector population tracking. Various trap types, such as Biogents Sentinel (BG) traps, Gravid *Aedes* Traps (GAT), Light Traps, and Ovitraps, have been deployed in GIS-mapped locations to monitor adult mosquitoes and egg-laying activity [40, 46, 48].

AI-integrated GIS systems are believed to enhance mosquito surveillance by automating the identification and classification of mosquito species from trap data. Machine-learning models trained on trap-derived data sets can predict mosquito abundance trends**,** providing early warnings for potential outbreaks [50]. Furthermore, integrating environmental parameters (temperature, humidity, wind speed, and rainfall) with GIS-based predictive modelling improves forecasting accuracy, allowing for proactive vector control. By leveraging GIS-driven spatial intelligence, dengue prevention programmes can maximise efficiency, reduce resource expenditure, and improve outbreak preparedness [51]. Combining mosquito trap data, spatial analysis, remote sensing, and AI-powered predictive modelling possibly enable sustainable and effective vector management, ultimately reducing dengue transmission and strengthening public health responses.

#### Urban and environmental planning for surveillance

Urbanisation, deforestation, and changing climate patterns have significantly influenced dengue transmission dynamics, making urban and environmental planning a key area, where GIS has been applied. Studies have demonstrated that poorly planned urban settlements, inadequate drainage systems, and increased population density create conditions conducive to dengue outbreaks [52].

In urban planning, GIS-based spatial analysis helps identify high-risk zones by integrating population density, temperature variations, and built-up areas [32]. Techniques such as MCDA and the analytical hierarchy process (AHP) prioritise areas requiring immediate intervention based on various urban risk factors [42]. KDE and hotspot analysis are applied to detect clusters of dengue cases, providing insights into spatial disease transmission patterns [38, 44]. Remote sensing and thermal imaging from satellites such as Landsat and Sentinel-2 have been employed to map land use patterns and urban heat islands, and environmental suitability modelling showing how temperature variations influence mosquito activity [30, 51, 53]. Spatiotemporal modelling using methods such as SaTScan allows the detection of emerging dengue hotspots over time, improving early response strategies [18]. GIS-integrated network analysis has also been utilised to optimise mosquito control logistics, such as mapping efficient insecticide spraying routes or placing vector control teams in the most affected urban regions [27, 28, 54].

Environmental GIS applications include advanced hydrological modelling using Digital Elevation Models (DEM) and watershed delineation to analyse flood-prone zones, where stagnant water promotes mosquito breeding [55, 56]. NDVI, Normalised Difference Water Index (NDWI) are widely used to identify areas with high vegetation and water accumulation, respectively, which serve as mosquito habitats. Topographic Wetness Index (TWI) and Soil Moisture Index (SMI) have been used to assess surface water retention capabilities, aiding in predicting potential breeding grounds [55, 57]. Spatial interpolation methods such as IDW and kriging help in predicting mosquito distribution based on past breeding site occurrences and environmental factors, such as humidity, rainfall, and land cover [28].

With the increasing availability of real-time environmental data, IoT-based sensor networks integrated with GIS platforms provide continuous updates on temperature, humidity, and rainfall, key factors in dengue risk prediction. Unmanned Aerial Vehicles (UAVs) or drone-based GIS mapping has further enhanced mosquito habitat detection by capturing high-resolution imagery of inaccessible breeding sites. These GIS-based approaches provide a comprehensive framework for surveillance, risk assessment, and proactive urban and environmental planning, ensuring better vector control and disease prevention strategies [10, 55].

#### Early warning systems (EWS) for dengue prediction

The use of GIS in EWS for dengue prediction has revolutionised outbreak forecasting by integrating spatiotemporal data, environmental parameters, and predictive modelling. Unlike traditional surveillance methods that rely on retrospective case data, GIS-based EWS proactively assesses dengue risk by analysing climatic factors (temperature, humidity, and rainfall), vector density indices, and urbanisation patterns to identify high-risk areas before outbreaks occur [58–60]. This predictive capability allows for timely interventions, resource optimisation, and data-driven decision-making, significantly improving dengue prevention and control strategies (Supplementary Table 1).

GIS enables spatial and statistical modelling of dengue transmission through various techniques. Geographically Weighted Regression (GWR), Bayesian Spatial Models, Generalised Linear Models (GLM), and Generalised Additive Models (GAM) help establish associations between dengue cases and environmental determinants, capturing localised variations in risk [61]. Machine-learning algorithms, such as random forest (RF), support vector machines (SVM), and deep learning models, such as long–short-term memory (LSTM), artificial neural networks (ANN), convolutional neural networks (CNN), and feedforward neural networks (FNN), leverage complex data sets to detect outbreak patterns with high accuracy and need more exploration [62–64]. In addition, time-series models such as auto-regressive integrated moving average (ARIMA) and distributed lag non-linear models (DLNM) assess the temporal impact of climate variability on dengue transmission, aiding in seasonal outbreak predictions [65, 66].

Spatial interpolation and cluster analysis within GIS further enhance the predictive potential of EWS. Techniques such as IDW and kriging estimate surveillance risk in unsampled areas by interpolating case distributions based on spatial proximity [67]. These methods transform raw epidemiological data into dynamic risk maps, providing public health agencies with real-time geospatial intelligence for outbreak management [49, 68]. Studies highlight that combining real-time GIS surveillance with predictive analytics has significantly improved the timeliness and accuracy of dengue forecasting, allowing governments and public health agencies to take pre-emptive action against outbreaks rather than reactive responses.

A total of *n* = 15 of studies focused on EWS for dengue prediction and outbreak risk assessment (Fig. 6B). The benefits of GIS-driven EWS in surveillance control are substantial. By integrating predictive analytics with real-time GIS surveillance, these systems facilitate early detection of outbreak-prone areas, enabling proactive mosquito control, optimised resource allocation, and precision public health measures. Visualising spatial risk maps through GIS dashboards empowers policymakers with actionable insights, ensuring a strategic and pre-emptive approach rather than a reactive response.

The future of GIS-based surveillance prediction lies in leveraging big data analytics, remote sensing technologies, and AI-driven forecasting models. By incorporating satellite-derived environmental monitoring, IoT-based vector tracking, and cloud-based GIS platforms, EWS can achieve higher accuracy, real-time adaptability, and enhanced outbreak preparedness. These advancements will be crucial in shifting surveillance from reactive to proactive, ultimately reducing disease burden and improving public health outcomes.

#### Disease surveillance and public health monitoring

GIS has transformed disease surveillance and public health monitoring by providing seasonal insights into the spatial spread of dengue. Among the reviewed studies, *n* = 14 focused on dengue surveillance and public health monitoring, highlighting the significance of GIS in tracking outbreaks, assessing risk factors, and improving response efficiency (Fig. 6B). Through advanced spatial epidemiological techniques and geostatistical analysis, such as choropleth maps, heat maps, and spatiotemporal cluster detection, researchers can visualise outbreak patterns, detect emerging hotspots, and implement timely interventions [35, 69, 70].

Mobile GIS applications enable real-time data collection through GPS-enabled devices and community reporting, improving surveillance in remote and underserved areas [71]. Cloud-based GIS platforms, including Google Earth Engine, ArcGIS Online, and QGIS, have enabled interactive mapping and dashboard-based disease tracking, allowing policymakers to assess outbreak severity, evaluate intervention effectiveness, and allocate resources efficiently. Integrating GIS with mobile applications, crowdsourced surveillance and participatory mapping has strengthened surveillance networks by leveraging real-time community reporting and health information systems [71, 72]. In addition, geostatistical methods such as kriging and IDW assist in predicting disease spread, particularly in data-scarce regions. Network analysis helps optimise healthcare accessibility by strategically placing hospitals, clinics, and emergency response services based on population density and transportation networks [73].

Integrating GIS with AI and machine learning can enhance predictive capabilities and outbreak forecasting, automate hotspot detection, improve spatial pattern recognition, and optimise vector control measures. AI-driven deep learning models and spatiotemporal analysis can refine outbreak predictions and provide data-driven decision support. Agent-based modelling (ABM) simulates disease transmission dynamics, while MCDA combines multiple health indicators to prioritise high-risk areas for intervention [11, 54].

Satellite and drone-based remote sensing enable continuous monitoring of environmental factors, such as vegetation, water stagnation, and urbanisation, which influence vector-borne disease transmission. Sensor-based GIS utilising IoT technology provides real-time environmental data, such as mosquito density, air quality, and water contamination levels, supporting proactive public health responses.

#### Resource allocation and healthcare planning

Ensuring efficient resource allocation and healthcare accessibility is another crucial application of GIS in dengue surveillance. Healthcare facilities must be strategically placed during outbreaks to provide timely diagnosis and treatment [74]. GIS-based accessibility models, such as network analysis and distance mapping, have been used to evaluate the proximity of affected populations to healthcare centres [17, 47, 54, 73]. In addition, location–allocation analysis helps determine the best locations for setting up temporary dengue treatment centres during peak outbreak periods [17].

One primary GIS technique in healthcare planning is network analysis, which evaluates travel times and distances between affected populations and healthcare centres. Network analysis is a method for understanding the interconnected factors contributing to dengue outbreaks. This helps identify underserved areas requiring additional facilities or mobile healthcare units [71, 75]. Distance mapping and service area analysis further assist in determining the accessibility of hospitals and clinics, ensuring that dengue patients receive timely diagnosis and treatment [17, 47, 54, 73]. Location–allocation modelling is another critical GIS technique used for optimising healthcare resource distribution. This method helps identify the best locations for setting up temporary dengue treatment centres, fever clinics, or diagnostic labs based on outbreak severity and population density. In addition, it aids in efficiently allocating hospital beds, medical supplies, and healthcare personnel by predicting high-risk zones that may experience a surge in dengue cases [76]. GIS-based hotspot analysis and spatial clustering techniques are used to map dengue case concentrations, allowing health authorities to deploy resources in a data-driven manner. By overlaying healthcare facility locations with dengue incidence maps, authorities can identify healthcare gaps and prioritise interventions in the most affected areas.

Mobile GIS applications and community-based participatory mapping have also improved surveillance response, particularly in rural and resource-limited regions [34, 71]. These applications enable real-time data collection on case reports, mosquito breeding sites, and healthcare facility capacity, enhancing outbreak management [71]. Furthermore, GIS-driven epidemiological models integrate environmental and demographic data to predict future outbreaks, helping in proactive healthcare planning.

By leveraging GIS, public health agencies can enhance disaster preparedness, ensuring that healthcare systems are equipped to handle dengue outbreaks efficiently. From optimising emergency response routes to predicting future healthcare demand, GIS provides a spatial intelligence framework that strengthens healthcare infrastructure and improves patient outcomes during epidemics.

### Variables used in the GIS-based dengue surveillance studies

**Fig. 7:**
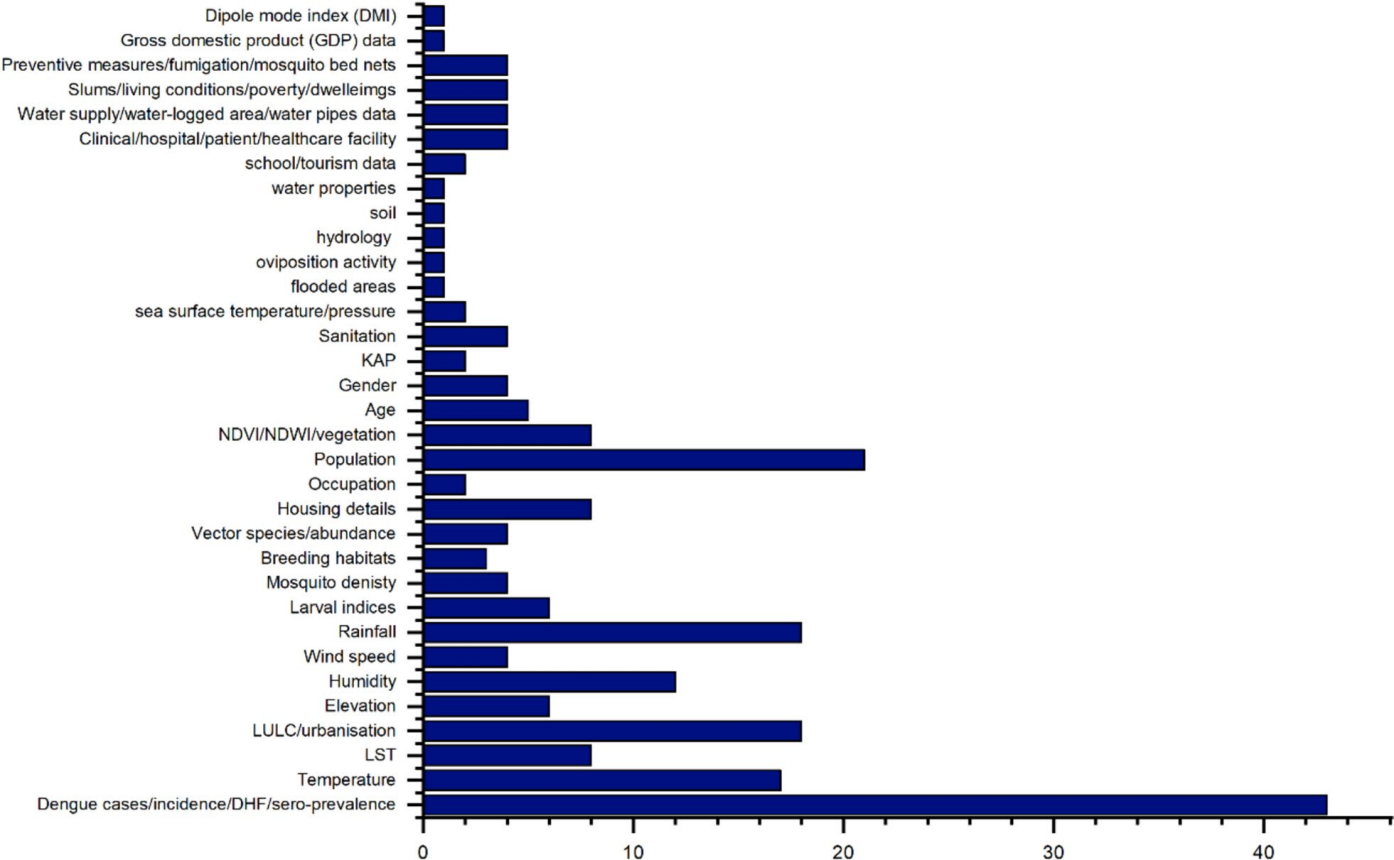
Study variables

The most frequently analysed key variables that occurred were dengue cases/incidence (*n* = 43), population data (*n* = 19), rainfall (*n* = 19), temperature (*n* = 18), humidity (*n* = 13), and LULC (*n* = 13), highlighting the strong emphasis on environmental and demographic risk factors, shown in Fig. 7. Other variables such as wind speed, NDVI, mosquito indices, and socio-economic indicators were used less frequently but provided critical insights into localised disease dynamics and vector ecology.

Several other variables remain underutilised despite their potential to enhance disease prediction and spatial modelling accuracy. Wind speed and direction are crucial in mosquito dispersion; although *Aedes aegypti* typically has a limited flight range, wind conditions can influence the passive movement of mosquitoes, expand their breeding territory and contribute to the spread of disease. Incorporating wind data into GIS models can support the development of mosquito dispersion maps and help anticipate outbreak zones based on prevailing wind patterns [50, 58].

Elevation and slope are additional variables with significant yet underexplored importance in disease modelling. These topographic factors influence temperature, humidity, and water flow, affecting vector habitat suitability. Low-lying areas with flat slopes are more prone to water stagnation, creating optimal breeding conditions for mosquitoes. DEMs, slope analysis, and hydrological modelling tools within GIS can, therefore, aid in identifying flood-prone or water-retaining regions, especially in peri-urban and rural landscapes [24, 42].

Similarly, soil moisture content and hydrological factors are valuable for predicting larval breeding grounds, particularly in areas lacking visible water bodies. Soil moisture supports the survival of eggs and larvae, especially for *Aedes* species that can breed in moist environments even without open water. Including such hydrological indicators in GIS-based studies can significantly improve the predictive accuracy of habitat models and inform more localised vector control interventions [57]. Water quality parameters, including pH, nutrient levels, and organic content, can also influence vector ecology. Studies have shown that mosquitoes prefer specific conditions for breeding, and polluted water with high nutrient content can often sustain higher larval densities. Future studies could enhance habitat risk models by incorporating these parameters, particularly in urban slums or industrial zones with poor sanitation [77].

Another variable is the urban heat island effect, where densely built-up areas trap heat and create warmer microclimates. These elevated temperatures can increase mosquito metabolism and accelerate virus replication cycles, leading to higher transmission risk. Remote sensing-derived LST and urban heat maps can be used in GIS to detect such microclimates and inform urban planning strategies to reduce heat pockets and disease risk [30, 51, 53]. Human mobility data, such as commuting patterns, migration flows, and tourism hotspots, are crucial yet rarely integrated into GIS-based surveillance. Unlike mosquitoes, infected individuals can carry the virus long distances, facilitating the introduction of the disease into new regions [78]. Incorporating mobile phone location data, transportation routes, and travel history into GIS models could significantly improve outbreak forecasting and help identify potential corridors of disease spread.

Socio-economic variables such as housing conditions, literacy levels, access to healthcare, and sanitation are fundamental determinants of vulnerability to vector-borne diseases [20, 70, 74]. Including these variables would allow researchers to assess health disparities, prioritise interventions in high-risk communities, and implement more equitable resource allocation strategies. Variables related to waste management infrastructure and drainage systems also offer significant value in GIS modelling. Poor waste disposal and blocked drainage systems contribute to mosquito breeding, especially in urban areas [9]. Mapping these infrastructures using GIS can support risk zoning and optimise the deployment of larval source management interventions.

### GIS modelling techniques used in dengue surveillance

**Fig. 8:**
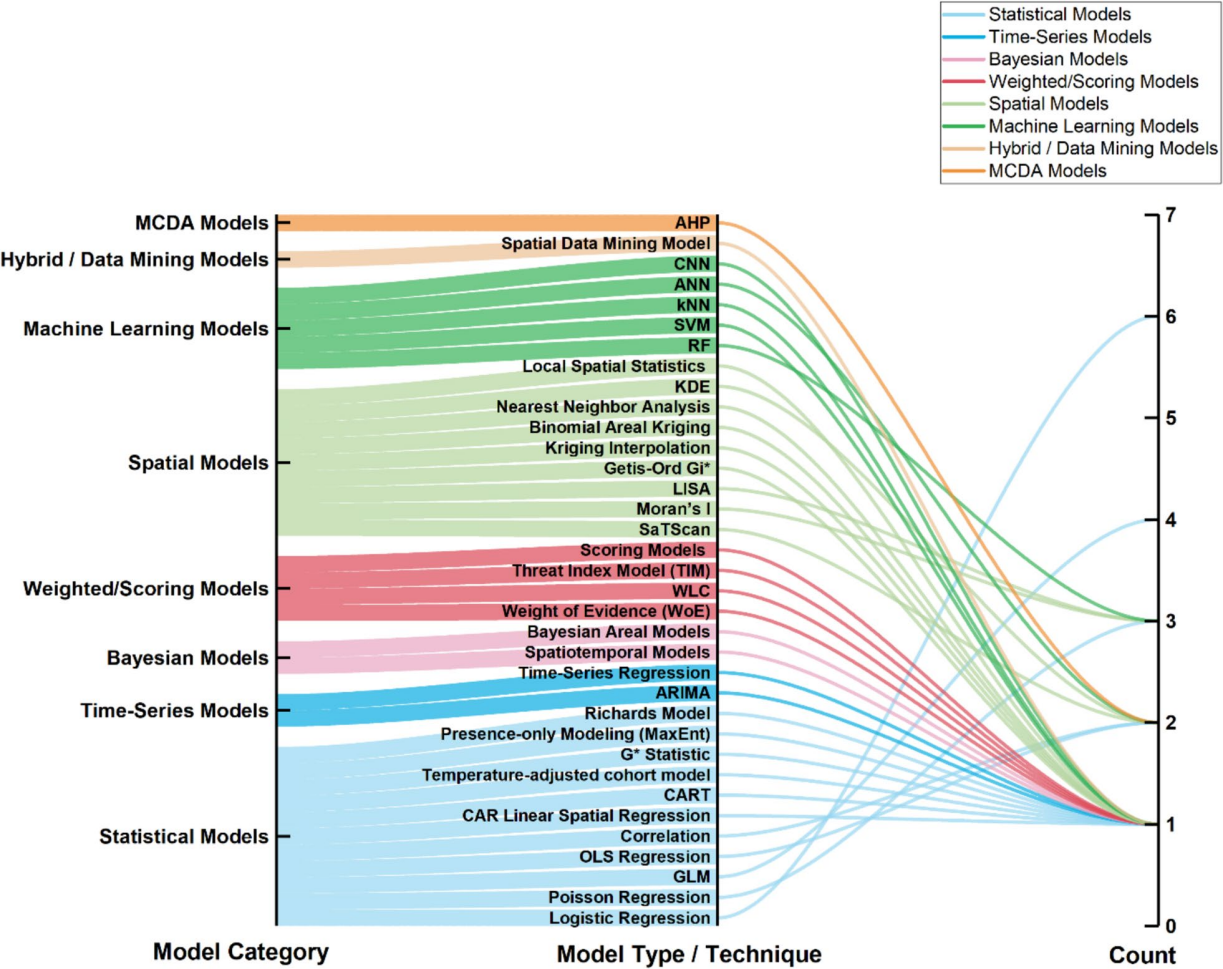
Modelling techniques used in studies for GIS-based dengue surveillance

Across the reviewed studies, various modelling techniques were employed to support GIS-based surveillance, grouped into eight distinct categories as visualised in Fig. 8. Statistical models emerged as the most widely used category, with logistic regression, ordinary least squares (OLS), and GLM frequently applied to examine associations between disease incidence and explanatory variables [25, 79, 80]. These models enabled researchers to identify significant environmental and socio-demographic disease transmission predictors and quantify risk relationships in spatially referenced data. Following statistical models, spatial models formed the second most common category. These included techniques, such as Moran’s I, LISA, Getis-Ord Gi, kriging, and SaTScan, which were primarily used for detecting spatial clustering, measuring spatial autocorrelation, and generating continuous surface maps of risk areas. Among these, Moran’s I and LISA were the most frequently used spatial statistics applied to assess the non-random spatial distribution of dengue cases [35, 37].

Other modelling approaches were less common but showed growing interest, particularly in recent studies. Machine-learning models, including RF, SVM, ANN, and CNN, have begun to appear more frequently, offering advanced predictive capabilities [10, 52, 81]. Weighted and scoring models, such as Weight of Evidence (WoE) and Threat Index Models (TIM), were used to generate risk maps through composite indicators [53, 60]. A smaller subset of studies used time-series models such as ARIMA and Richards models to forecast disease trends [23, 66]. Bayesian models were applied for high-resolution risk estimation, including areal and spatiotemporal formulations [29]. MCDA models, such as the AHP, were used in studies aiming to prioritise intervention zones based on environmental and socio-economic criteria [9, 42].

## Discussion

Before the adoption of GIS, dengue surveillance primarily relied on manual data collection, paper-based mapping, and basic statistical summaries at aggregated administrative levels. These traditional methods lacked spatial precision, often led to delayed outbreak detection, and were limited in their ability to visualise or predict the spread of disease in real time. Epidemiological data were generally analysed using descriptive statistics or tabular formats, which did not capture spatial heterogeneity or environmental risk factors influencing mosquito breeding and disease transmission. With the growing complexity of urban environments and climate variability, such conventional approaches proved insufficient for proactive surveillance and control. The integration of GIS has addressed many of these gaps by enabling real-time mapping, spatial clustering, risk zoning, and environmental modelling. GIS tools allow for the layering of epidemiological, demographic, and climatic data, thus supporting more targeted vector control, timely outbreak responses, and informed resource allocation. Advanced GIS platforms support the use of remote sensing, spatial statistics, and ML/AI, making surveillance systems more robust and predictive [82, 83].

A comparative analysis of recent reviews highlights the fragmented role of GIS in dengue surveillance. Prior studies emphasised the importance of translating geospatial data into actionable public health strategies and fostering intersectoral collaboration; other reviews focused on spatial risk assessments using remote sensing, spatial interpolation, and regression modelling [84, 85]. Other reviews showcased GIS integration with remote sensing and multi-criteria decision analysis for dengue risk mapping based on environmental and demographic predictors [86, 87]. However, most lacked a comprehensive framework, overlooking behavioural and social determinants and offering limited thematic categorisation. This scoping review addresses these gaps by presenting a multifocal synthesis of GIS applications across six public health domains: risk mapping, early warning systems, vector control, disease surveillance, urban planning, and healthcare resource allocation. It systematically compares application frequencies, regional adoption, and emerging trends, highlighting critical gaps, such as the underutilisation of participatory GIS and AI/ML tools.

This scoping review demonstrates its effectiveness in mapping vector distribution, identifying hotspots, and integrating environmental and climatic factors to enhance public health decision-making. Most studies focused on risk mapping and hotspot identification (*n* = 26), highlighting its critical role in dengue surveillance. Using interpolation methods such as IDW and kriging also provided insight into the spatial distribution of dengue cases and vector indices. These methods help identify spatial clustering patterns, which can be further correlated with environmental variables and applied to many fields of infectious disease [88, 89]. Disease surveillance and public health applications were also widely studied (*n* = 14), followed by vector control and monitoring (*n* = 23). Applications related to resource allocation and healthcare (*n* = 11) and urban and environmental planning (*n* = 17) were less frequently explored. EWS accounted for *n* = 15 of the studies, indicating a growing but relatively lower emphasis on predictive modelling for surveillance outbreaks. Across the 64 included studies, GIS emerged as a critical enabler of spatial intelligence, allowing for precise mapping of disease incidence, vector habitats, and environmental risk zones and facilitating data-driven public health interventions.

The predominance of studies from South and Southeast Asia, particularly India, Thailand and Indonesia, emphasises the urgent need for geospatial tools in endemic regions, where outbreaks pose significant public health threats. GIS-based mapping should bridge human, animal, and environmental health. Beyond traditional epidemiological approaches, integrating GIS with “One Health” frameworks could possibly help identify overlooked reservoirs or alternative hosts in dengue transmission, expanding our understanding of disease ecology.

A significant proportion of studies incorporated environmental variables, particularly temperature and rainfall, to understand their influence on dengue transmission. The findings suggest that temperature and rain play a crucial role, and positive correlations in mosquito abundance and disease transmission were observed [90]. Apart from the basic environmental variables, such as temperature, humidity, and rainfall, additional factors such as wind speed and direction, pH, container depth, and water volume, and water nutrients influence the growth of larvae can provide a more comprehensive understanding of mosquito breeding patterns [15, 91] Recent advancements in bioacoustics monitoring suggest that mosquito species activity can be detected based on their wingbeat frequency. GIS-integrated sound sensors in urban areas could help map high mosquito density zones, offering a novel, non-invasive surveillance method that enhances real-time vector monitoring [92]. Integrating satellite imagery, remote sensing, and climate variables has further improved the accuracy of vector habitat identification and risk prediction by EWS and modelling. Machine-learning approaches, such as RF and regression models, have been increasingly applied to predict dengue outbreaks [52, 66, 81]. These models integrate spatial and non-spatial data to improve EWS, helping public health authorities allocate resources more effectively. AI-driven GIS models and drone-based surveillance data may guide resource allocation, ensuring that medical supplies, vector control teams, and rapid response units are distributed efficiently based on real-time risk assessment.

Remote sensing allowed researchers to assess land use patterns, identify potential mosquito breeding sites, and develop risk maps. NDVI and LULC classification were common predictors identified, which have provided insights into the influence of vegetation and water bodies on vector proliferation [26, 31]. Urbanisation and the heat island effect may create microclimates favouring mosquito survival and virus transmission. GIS-based studies should integrate urban thermal and vulnerability mapping techniques to assess localised temperature variations and their impact on dengue incidence [43].

A key insight from the review is that statistical modelling techniques remain the most employed approach in GIS-based dengue surveillance studies. These include regression-based analyses such as logistic, Poisson, and generalised linear models, effectively quantifying relationships between disease incidence and various environmental and demographic risk factors [79]. Their wide application reflects their ease of implementation, interpretability, and compatibility with spatial and non-spatial data sets. Spatial models were the next most frequently used category and played a central role in identifying the geographic clustering of disease cases. Techniques such as Moran’s I, LISA, and Getis-Ord Gi provided critical insights into spatial autocorrelation and hotspot dynamics, enabling targeted intervention strategies [93]. While spatial models enhance visual understanding and pattern detection, statistical modelling frameworks significantly strengthen their interpretive power. Machine-learning models have recently gained momentum in dengue modelling due to their ability to handle complex, high-dimensional data sets and uncover non-linear patterns [10]. Including MCDA frameworks and hybrid geostatistical models further supports a growing trend towards interdisciplinary and integrative modelling approaches that combine spatial, environmental, and behavioural data for comprehensive risk assessment [9].

The findings highlight the importance of spatial analysis techniques in identifying high-risk areas and the need for interdisciplinary approaches to integrate environmental, climatic, and socio-economic factors, such as human migration, human behaviour, and population growth. Tourism data can also be included in GIS-based dengue surveillance to assess disease risk in high-traffic areas, helping authorities implement targeted prevention measures and protect travellers and local populations [94]. Advancements in GIS technology and improved data integration will be critical in strengthening dengue prevention and control strategies globally. This GIS-based approach is highly adaptable and can be applied to analyse any spatial data related to any infectious diseases, enabling comprehensive monitoring, risk assessment, and targeted intervention strategies for various public health challenges [95].

Most studies focus on environmental factors and landscape patterns; human mobility patterns, including daily commuting, migration, and tourism, are critical in shaping dengue transmission dynamics [94]. Integrating mobile phone data, Geographic positioning systems (GPS) tracking, and transportation network analysis into GIS models can enhance outbreak predictions by capturing real-time movement trends. National boundaries do not confine dengue transmission; most systems operate within administrative limits. Establishing cross-border GIS frameworks could improve disease tracking, particularly in high-risk regions near international borders and global travel hubs.

The emergence of smart cities, where urban infrastructure integrates technology and real-time data, presents new opportunities for surveillance. Embedding GIS-based surveillance models into innovative waste management, IoT-based mosquito monitoring systems, and intelligent water distribution networks could proactively mitigate breeding grounds. Drone-assisted GIS, leveraging high-resolution multispectral cameras, offers a novel approach for mapping stagnant water bodies, identifying high-risk zones, and deploying larvicides in inaccessible areas, warranting further research.

Public perception, misinformation, and behavioural resistance, such as reluctance to use repellents or scepticism towards vaccines, can significantly influence disease outbreak dynamics. GIS-based behavioural mapping can help identify regions, where targeted health interventions and awareness campaigns are necessary [96].

Importantly, the GIS-based approach is not limited to surveillance but is a universal tool for tracking and managing various infectious diseases, allowing for improved spatial epidemiology, rapid outbreak response, and enhanced public health decision-making on a global scale. These advancements emphasise the need for interdisciplinary research integrating GIS, artificial intelligence, and emerging technologies to strengthen global surveillance and control strategies. While GIS provides powerful spatial insights, its value is maximised when integrated into broader disease surveillance frameworks. The insights from GIS mapping, such as identifying spatial clusters, environmental risk zones, or underserved areas, can directly inform public health decision-making, health education campaigns, and urban planning. By aligning GIS-based insights with epidemiological data, surveillance systems can transition toward integrated, multidisciplinary approaches that address both environmental and social determinants of disease. This highlights the need for cross-sector collaboration between public health authorities, urban planners, and data scientists.

**Challenges faced in the public health sector for disease surveillance**

- Integration of multi-source data
  Effective GIS-based surveillance requires the seamless integration of health data, climatic variables, remote sensing imagery, and demographic information. This necessitates standardised data formats and inter-agency coordination, which is often lacking in practise.
- Technical expertise and infrastructure gaps
  Many public health institutions in endemic regions lack trained GIS professionals and the required infrastructure, posing barriers to full-scale implementation.
- Ethical and privacy concerns
  The use of geospatial health data raises ethical challenges. Mapping cases to specific locations may inadvertently reveal private health information, raising concerns about patient confidentiality and potential stigmatisation of communities.
- Institutional and policy barriers
  Fragmented health data systems, unclear data-sharing protocols, and limited financial resources hinder the institutionalisation of GIS in routine public health workflows.
**Limitations of GIS tools**
- Data availability and quality
  Many studies reported inconsistent, incomplete, or low-resolution epidemiological and environmental data sets. These limitations directly affect the accuracy and reliability of spatial models and risk predictions.
- Temporal limitations
  Most reviewed studies relied on retrospective and cross-sectional data, which restricts the development and application of real-time surveillance or early warning systems. The absence of near-real-time data hampers proactive decision-making.
- Analytical complexity and interoperability
  The integration of GIS with statistical models, remote sensing data, and machine-learning techniques often requires advanced computational capacity and interoperability between platforms, which is technically demanding.
- Spatial resolution and bias
  GIS models are sensitive to the resolution of the spatial layers used. Coarse data can obscure micro-level patterns of transmission, while biased spatial inputs (e.g., under-reporting from rural areas) may skew results.

## Future directions and recommendations

Future research should integrate multi-dimensional data sets including socio-demographic, patient health (e.g., co-morbidities), and mobility data to enhance understanding of dengue transmission dynamics and climate-related shifts in vector habitats. Longitudinal GIS modelling can support forecasting mosquito breeding changes due to evolving weather patterns, aiding policy planning.

To advance GIS-based dengue surveillance, the following recommendations are proposed:

- Enhance real-time surveillance: Incorporate mobile GIS, remote sensing, and IoT technologies for dynamic, timely outbreak detection and response.
- Leverage AI and machine learning: Integrate AI/ML with GIS to improve predictive accuracy, hotspot identification, and automated risk mapping.
- Strengthen interdisciplinary collaboration: Facilitate partnerships among epidemiologists, data scientists, urban planners, and policymakers to develop effective and scalable GIS-based strategies.
- Improve data standardisation and accessibility: Establish centralised, interoperable, and open-access GIS databases to enhance data quality, comparability, and transparency across studies.

## Conclusion

This scoping review emphasises the transformative role of GIS in dengue surveillance, providing essential spatial intelligence for public health decision-making from risk mapping and early warning systems to vector monitoring and urban planning. The integration of GIS with ML and AI presents a powerful opportunity to shift from reactive responses to predictive surveillance. ML algorithms can model complex, non-linear interactions among environmental, climatic, and demographic variables, significantly improving the accuracy of risk forecasts. AI-enabled systems can automate hotspot detection, enhance outbreak prediction, and support real-time decision-making through mobile GIS platforms. In practical settings, health departments can leverage these technologies for early warnings, dynamic resource allocation, and geographically targeted interventions. Further advancement requires interdisciplinary collaboration, integration with IoT-based sensor networks, and the development of participatory GIS platforms to ensure more intelligent, scalable, and proactive surveillance systems not only for dengue but also for broader public health challenges or any disease surveillance.

## Supplementary Information

Supplementary Material 1.

## Data Availability

No data sets were generated or analysed during the current study.

## Abbreviations

VBDs: Vector-borne diseases
GIS: Geographical information system
PRISMA-ScR: Preferred Reporting Items for Systematic Reviews and Meta-Analyses Extension for Scoping Reviews
NDVI: Normalised Difference Vegetation Index
AI: Artificial intelligence
IoT: Internet of things
ML: Machine learning
WHO: World Health Organisation
LULC: Land use land cover
MCDA: Multi-criteria decision analysis
EWS: Early warning systems
MODIS: Moderate resolution imaging spectroradiometer
LST: Land surface temperature
LISA: Local Indicators of Spatial Association
KDE: Kernel density estimation
IDW: Inverse distance weighting
SaTScan: Spatial scan statistics
STC: Spatiotemporal clustering
PPA: Point pattern analysis
HI: House index
BI: Breteau index
CI: Container index
GAT: Gravid *Aedes* traps
BG: Biogents sentinel
AHP: Analytical hierarchy process
DEM: Digital elevation model
NDWI: Normalised difference WATER Index
TWI: Topographic Wetness Index (TWI)
SMI: Soil Moisture Index
UAVs: Unmanned aerial vehicles
GWR: Geographical weighted regression
GAM: Generalised additive model
GLM: Generalised linear model
OLS: Ordinary least square
RF: Random forest
SVM: Support vector machine
LSTM: Long–short-term memory
CNN: Convolutional neural networks
ANN: Artificial neural network
FNN: Feedforward neural networks
DLNM: Distributed lag non-linear models
AGM: Agent-based model
TIM: Threat index model
WoE: Weight of evidence
ARIMA: Auto-regressive integrated moving average
