# Supplementary material for: Leveraging geographic information system for dengue surveillance: a scoping review
Source: Trop Med Health. 2025 Aug 4;53:102. doi: 10.1186/s41182-025-00783-9 (PMC12320306; doi:10.1186/s41182-025-00783-9)
Supplement: Supplementary file 1 — Supplementary Material 1. [file 41182_2025_783_MOESM1_ESM.docx]

**Table 1: Multifocal applications of GIS-based dengue surveillance studies**

| **Study ID** | **Author/s** | **Year** | **Study area** | **Dengue surveillance focus** | **Applications** |
| --- | --- | --- | --- | --- | --- |
| 1 | N. Bharathi et al (22) | 2015 | India | Investigating risk factors, prevalence, and incidence of dengue and chikungunya | Risk mapping, disease surveillance, public health planning |
| 2 | Markdy Y. Orong et al (66) | 2015 | Philippines | Forecasting dengue outbreaks from 2016 to 2020 using GIS and ARIMA | Aids in forecasting dengue outbreaks and implementing preventive measures |
| 3 | Soumendranath Chatterjee et al (15) | 2015 | India | Monitoring mosquito populations, breeding sites, and environmental factors | Management of vector control programs by targeting specific habitats based on water quality parameters, to predict outbreaks and guide control measures |
| 4 | B N Nagpal et al (16) | 2015 | India | Monitoring *Aedes* mosquito populations, identifying breeding sites, tracking dengue case trends | Continuous surveillance and control efforts in managing *Aedes* mosquito populations and preventing dengue outbreaks |
| 5 | David F. Attaway et al (21) | 2016 | Africa | Identifying high-risk areas | Risk mapping, public health planning, climate change modelling |
| 6 | Shahbaz Ahmad et al (82) | 2016 | Pakistan | To map dengue's temporal changes and assess regional burden | Monitoring disease patterns, identifying high-risk areas, for effective surveillance and risk identification |
| 7 | Mohd Hazrin et al (39) | 2016 | Malaysia | To map and analyse the spatial distribution of dengue cases in Putrajaya using GIS and spatial statistical analyses. | Identify dengue hotspots, guide control measures, and inform public health planning and interventions |
| 8 | Pitcha Ratanawong et al (83) | 2016 | Thailand | Focused on spatial patterns of dengue transmission in schools and households in rural, semi-rural, and semi-urban areas of Thailand | Targeted vector control, policy recommendations, entomological monitoring |
| 9 | Manuel O Espinosa et al (84) | 2016 | Argentina | Space-time dynamics of *Aedes aegypti* breeding sites in Clorinda City, Argentina, and their association with environmental factors to develop a dengue vector niche model. | Development of risk maps and modelling for targeted vector control strategies, informing urban planning policies to mitigate breeding site risks |
| 10 | Nadeem Fareed et al (60) | 2016 | Pakistan | Using GIS and RS to analyse dengue transmission patterns across two regions in Pakistan | EWS for dengue outbreaks, risk mapping for targeted vector control, urban planning to minimize breeding sites, climate and health policy integration |
| 11 | PDNN Sirisena et al (6) | 2017 | Sri Lanka | Mapping and evaluating the spatial and temporal distribution of dengue, analysing its association with climatic | Identifying high-risk areas, forecast future outbreaks, RS enhanced the monitoring of climatic factors relevant to dengue |
| 12 | Aileen Kenneson et al (86) | 2017 | Ecuador | To examine household and community level risk factors and assessing the impact of Knowledge, Attitudes, and Practices (KAPs) and socio-ecological factors on household | Targeted vector interventions, guiding community-level programs for garbage collection and cleanup of abandoned properties. |
| 13 | Maria F. Vincenti-Gonzalez et al (69) | 2017 | Venezuela | Identifying dengue hot spots and assessing risk factors associated with clusters | Can guide targeted dengue surveillance and control efforts, especially in high-risk zones, provides a framework for more efficient use of public health resources in dengue control |
| 14 | Daniel Adyro Martínez-Bello (29) | 2017 | Colombia | Bayesian disease mapping for dengue risk estimation at a small spatial scale. | Improved spatial risk mapping for targeted vector control, integration of satellite data into dengue early warning systems. |
| 15 | Tanawat Chaiphongpachara et al (90) | 2017 | Thailand | DHF risk assessment in a tourist destination. | Public health planning, dengue prevention, tourist safety measures. |
| 16 | Shuchi Mala and Mahesh Kumar Jat (52) | 2018 | India | Predicting high-risk areas for dengue by integrating meteorological parameters, ANN, and GIS to develop a spatial data mining model. | Aids public health officials and policymakers in reducing dengue risk, particularly in rural and urban areas of Delhi |
| 17 | Masroor Ali Khan et al (50) | 2018 | Saudi Arabia | To assess the relationship between climatic variables and mosquito abundance, particularly *Aedes* mosquitoes. | Predicting vector-borne disease risks, and planning targeted interventions for mosquito control |
| 18 | Carbajo, Aníbal E et al (87) | 2018 | Argentina | To study spatial and demographic analysis of dengue cases, particularly distinguishing between imported and autochthonous cases, during the largest outbreak in 2016. | Vector control strategies, prioritizing high-risk areas, and understanding how demographic factors contribute to the spread of dengue. |
| 19 | Yujuan Yue et al (25) | 2018 | China | Investigating the spatial patterns of dengue fever considering environmental and socio-economic risk factors at a fine spatial scale | Model helps to identify spatial clusters, inform public health strategies for targeted prevention and control measures |
| 20 | Daniel Martínez-Bello et al (26) | 2018 | Colombia | Developing spatiotemporal models to estimate the relative risk of dengue | The models generate relative risk maps, which can identify high-risk areas, improve public health surveillance and forecasting. |
| 21 | Mattia Sanna et al (23) | 2018 | China | To understand spatial-temporal progression, identify hotspots, and evaluate mitigation efforts | Early hotspot detection, targeted interventions, and improving outbreak response strategies |
| 22 | Janet Ong et al (81) | 2018 | Singapore | Stratifying spatial risk of dengue transmission for efficient resource allocation in Singapore | Provides a reliable dengue risk map to guide vector control strategies, enabling effective allocation of resources in high-risk areas |
| 23 | Gayan P. Withanage et al (58) | 2018 | Sri Lanka | Developing and validating a weather-based forecasting model for early warning of dengue outbreaks in the Gampaha, Sri Lanka. | EWS for dengue outbreaks, targeted vector control and intervention planning, hospital resource allocation and preparedness, climate driven disease surveillance integration. |
| 24 | Sk Ajim Ali & Ateeque Ahmad (40) | 2018 | India | AHP and GIS for dengue risk mapping. Integration of environmental parameters to identify potential risk areas and development of a Spatial Decision Support System (SDSS). | Urban health planning, resource allocation for mosquito control programs, Early warning system development for dengue outbreaks. |
| 25 | Shahbaz Ahmad et al (7) | 2018 | Pakistan | To identify dengue hotspots and seasonal trends | Identify dengue hotspots and optimize control measures, informed decision making for public health strategies, including targeted vector control efforts. |
| 26 | Krishna Sankar Ganguly et al (35) | 2018 | India | Spatial clustering and spread of dengue in Kolkata. | Policymaking, urban health planning, predictive modelling. |
| 27 | Suchi Mala & Mahesh Kumar Jat (33) | 2019 | India | Investigating spatial and temporal distribution | Identifying high-risk areas for DF outbreaks, understanding the role of environmental factors |
| 28 | Muhammad A. Butt et al (31) | 2019 | Pakistan | Mapping the spatial distribution of dengue outbreaks and identifying environmental factors that influence its spread. | GIS and environmental factors can be used in vector control strategies and early warning systems for dengue outbreaks |
| 29 | Sk Ajim Ali & Ateeque Ahmad (9) | 2019 | India | Integrating multi-criteria decision-making (MCDM) with GIS and RS to identify and map dengue-susceptible areas, considering multiple factors like environment, demography, breeding sites, and disease history. | Identification of high-risk zones for targeted vector control measures, provides a framework for incorporating environmental, demographic, and epidemic factors in disease surveillance, useful for urban planning and waste management to reduce vector-borne disease risks. |
| 30 | Irene Casas, Eric Delmelle (74) | 2019 | Colombia | Health facility utilization patterns during the 2010 dengue epidemic in Cali, Colombia | Resource allocation, prevention campaigns, healthcare planning |
| 31 | A. Sajeli Begum et al (44) | 2019 | India | Entomological survey of the *Aedes* *aegypti* mosquito, its breeding sites, and the relationship between container types | Identifying high-risk areas for dengue outbreaks by pinpointing positive houses and breeding sites, aids in efficient vector control |
| 32 | Jailos Lubinda et al (49) | 2019 | Mexico | Geographic distribution and environmental suitability modelling of *Aedes* mosquitoes | Enhances mosquito surveillance in areas with reported arbovirus cases |
| 33 | Jaisankar Ramasamy et al (89) | 2019 | India | Develop a spatial-temporal surveillance system for detecting dengue outbreaks in Tamil Nadu. | Enhanced outbreak detection through spatial-temporal modelling. Better visualization of high-risk areas for targeted vector control. Improved dengue surveillance strategies using geospatial analysis. |
| 34 | Sumiko Anno et al (59) | 2019 | Taiwan | Development of an early warning system (EWS) for dengue fever outbreaks. | Early warning systems for dengue outbreaks, predictive modelling. |
| 35 | Wongpituk K. et al (72) | 2020 | Thailand | Investigating DHF incidence and developing a GIS-based surveillance system with community participation. | Developing a surveillance system, risk mapping, and identifying areas at high risk for DHF outbreaks |
| 36 | Cipta Estri Sekarrini (41) | 2020 | Indonesia. | Vulnerability mapping of DHF using GIS | Disease risk assessment and resource allocation |
| 37 | Damião da Conceição Araújo et al (20) | 2020 | Brazil | To analyse the spatial distribution of dengue and its relationship with social inequalities using spatial modelling | Planning intersectoral public health policies, targeting vector control measures in areas with high social inequality. |
| 38 | Petrúcio Luiz Lins de Morais et al (19) | 2020 | Brazil | Analysing dengue incidence and its spatial distribution to understand how local climate conditions and population density affect dengue outbreaks. | Aids in understanding disease dynamics in cool climate cities, targeted prevention and control measures |
| 39 | Veerasak Punyapornwithaya et al (18) | 2020 | Thailand | To analyze the spatio-temporal distribution of dengue cases in Chiang Mai, Thailand, during the 2013 outbreak. | Early detection of outbreak clusters for better resource allocation, improved surveillance and forecasting systems, data driven public health strategies for dengue control in urban areas. |
| 40 | Waqas Shabbir et al (79) | 2020 | Pakistan | Identifying spatial and temporal trends of dengue outbreaks in Pakistan and understanding significant environmental and infrastructural factors influencing dengue prevalence. | Dengue risk mapping for targeted interventions, incorporation of climate based forecasting models for early outbreak warnings, urban planning to minimize breeding habitats in densely populated areas. |
| 41 | Md. Siddikur Rahman et al (10) | 2021 | Thailand | Mapping and predicting the abundance of the dengue vector, *Aedes aegypti* | GIS and machine learning models for spatial distribution mapping and predictive modelling of dengue vectors to inform control measures and early warning systems for dengue prevention |
| 42 | Yibin Zhou et al (46) | 2021 | China | Dengue vector surveillance using mosquito oviposition traps (MOT) and GIS-based spatial analysis to improve the monitoring of *Aedes albopictus* distribution and abundance. | Targeted vector control, temporal monitoring of *Aedes* *albopictus* populations, hotspot identification for resource allocation |
| 43 | Nuriah Abd Majid et al (45) | 2021 | Malaysia | Understanding the spatial distribution and clustering of dengue cases from 2014–2018 in Bangi District, Malaysia, to identify hotspots and trends | Assists in identifying dengue hotspots for targeted interventions, provides data for public health planning and vector control strategies, facilitates the development of predictive models for outbreak management |
| 44 | Heni Prasetyowati et al (34) | 2021 | Indonesia | Impact of climate variability on dengue fever incidence in Jakarta, Indonesia, with an emphasis on identifying high-risk areas and understanding the socio-demographic and ecological factors contributing to DF clustering | Focus on high-risk populations and hotspot areas identified through spatial clustering and CART analysis, public health strategies, resource allocation |
| 45 | Tsheten Tsheten et al (85) | 2021 | Bhutan | Analysing spatiotemporal patterns of dengue cases across subdistricts | Enhancing early detection systems, informing preparedness and timely intervention strategies during outbreaks |
| 46 | Felipe Dzul-Manzanilla et al (43) | 2021 | Mexico | The focus is on identifying and prioritizing persistent *Aedes* borne disease hotspots | Guides targeted surveillance and vector control in high-risk areas, prioritizing persistent disease hotspots. |
| 47 | Fatima Ibrahim Abdulsalam et al (61) | 2021 | Thailand | Understanding the relationship between weather variables and dengue transmission in Nakhon Si Thammarat, Thailand. | Risk mapping for targeted interventions, climate-informed vector control strategies. |
| 48 | Lia Faridah et al (38) | 2021 | Indonesia | Spatial pattern analysis of dengue outbreaks and demographic distribution in Bandung. | Can inform dengue control program planning and resource allocation, guiding interventions in identified high-risk areas. |
| 49 | Mohamad Hizham Mohamad Hanapi & Farah Ayuni Shafie (51) | 2021 | Malaysia | Spatial-temporal patterns, environmental influences, outbreak prediction. | Public health decision-making, outbreak forecasting, vector control strategies. |
| 50 | Daira N Abán Moreyra et al (28) | 2022 | Argentina | Monitoring *Aedes aegypti* oviposition activity | Spatiotemporal analysis of vector distribution, evaluation of interventions, and for vector control planning |
| 51 | Muhammad Aidil Roslan et al (48) | 2022 | Malaysia | Monitoring and analysing the spatial distribution and temporal patterns of *Aedes albopictus* mosquitoes | Guide to public health agencies in implementing more effective mosquito control strategies and identifying high-risk areas for dengue transmission |
| 52 | Ummyatul Hajrah et al (17) | 2022 | Indonesia | Monitoring cases, tracking mosquito populations, mapping hotspots, analysing environmental factors, and predicting outbreaks | Support for developing an early warning system for dengue outbreaks, identification of endemic zones to focus vector control and public health interventions |
| 53 | Mohiuddin Sharif et al (37) | 2022 | Bangladesh | Analysing spatial relationships between *Aedes aegypti* vector density and dengue case distribution | Dengue surveillance, vector control, public health planning, supports the allocation of resources for dengue prevention and control |
| 54 | John Robert C. Medina et al (32) | 2023 | Philippines | Spatio-temporal distribution, cluster and hotspot identification | Targeted dengue interventions, resource allocation, |
| 55 | Nathaniel Vincent A. Lubrica et al (55) | 2023 | Philippines | Monitoring environmental factors, demographic data, and spatial patterns using GIS | Spatial mapping, policy development, and targeted disease control measures |
| 56 | T.T.P. Jayadas et al (27) | 2023 | Sri Lanka | Identifying high-risk dengue areas in Jaffna district by analysing the influence of land-use patterns, socio-economic, and demographic factors, alongside reported dengue cases and serotypes in 2019. | The risk maps enable health authorities to identify and prioritize high-risk areas for targeted vector control measures. |
| 57 | Priya Verma et al (88) | 2023 | India | To study the impact of the COVID-19 pandemic on dengue, the genomic diversity of the virus, and its relationship with changing environmental conditions | Public health monitoring, surveillance and risk analysis |
| 58 | Amir Zakuwan Mohammad Foad et al (36) | 2023 | Malaysia | Analysing the spatial and temporal distribution of dengue fever to identify hotspots, detect patterns, and improve detection and containment of dengue outbreaks | Identifies areas at high risk of dengue outbreaks, temporal monitoring, public health planning |
| 59 | A S M Maksud Kamal et al (30) | 2023 | Bangladesh | Investigating the spatial relationship between urban environmental components and dengue incidence in Dhaka in 2019. | Risk mapping and hotspot identification can be used for dengue surveillance in other cities with similar urban and climatological characteristics. |
| 60 | Eggy Arya Giofandi et al (53) | 2024 | Indonesia | To identify high-risk mosquito environmental zones linked to dengue haemorrhagic fever transmission in Pekanbaru City, Indonesia | Development of spatially targeted vector control strategies in high-risk zones, early intervention and risk assessment for dengue prevention. |
| 61 | Norie Neil C. Acosta & Nelda A. Nacion (68) | 2024 | Philippines | Analyse the spatiotemporal patterns of dengue cases to identify hotspots and cold spots | Public health interventions, target emerging hotspots, resource allocation, policy formulation |
| 62 | Eggy Arya Giofandi et al (70) | 2024 | Indonesia | Understanding the spatial pattern of DHF and estimating the potential spread of outbreaks | Can guide targeted vector control efforts and resource allocation |
| 63 | Rian Nurtyawan & Derry Budiman (24) | 2024 | Indonesia | Mapping dengue fever distribution in Bandung City, Indonesia, using GIS and remote sensing. Assessing dengue vulnerability based on environmental parameters. | Urban planning for dengue risk reduction, targeted vector control and surveillance strategies in high-risk areas. |
| 64 | Samphutthanont, R. et al (42) | 2024 | Thailand | Investigating the spatial and temporal distribution of dengue fever in Phuket Province and understanding the spatial relationship with the *Aedes* mosquito larvae index. | To detect dengue hotspots and prioritize control efforts, findings can guide the planning of dengue fever prevention measures and inform policies in areas with similar land use patterns. |
